# First report from Bangladesh on genetic diversity of multidrug-resistant *Pasteurella multocida* type B:2 in fowl cholera

**DOI:** 10.14202/vetworld.2021.2527-2542

**Published:** 2021-09-26

**Authors:** Otun Saha, M. Rafiul Islam, M. Shaminur Rahman, M. Nazmul Hoque, M. Anwar Hossain, Munawar Sultana

**Affiliations:** 1 Department of Microbiology, University of Dhaka, Dhaka-1000, Bangladesh; 2 Department of Gynecology, Obstetrics and Reproductive Health, Bangabandhu Sheikh Mujibur Rahman Agricultural University, Gazipur-1706, Bangladesh; 3 Vice-Chancellor, Jashore University of Science and Technology, Jashore-7408, Bangladesh.

**Keywords:** biofilm formation, Fowl cholera, genotype B:2, multidrug-resistance, *Pasteurella multocida*

## Abstract

**Background and Aim:**

Fowl cholera (FC) caused by *Pasteurella multocida* is a highly contagious bacterial disease of global importance for poultry production. The severity and incidence of FC caused by *P. multocida* may vary considerably depending on several factors associated with the host (including species and age of infected birds), the environment, and the bacterial strain. This study aimed to investigate the genetic diversity of multidrug-resistant *P. multocida* strains isolated from FC outbreaks in laying hens from commercial farms of Bangladesh.

**Materials and Methods:**

We collected 57 samples of suspected FC, including 36 live and 21 dead laying hens. *P. multocida* isolates were characterized by biochemical and molecular-biological methods.

**Results:**

Twenty-two strains of *P. multocida* were isolated from these samples through phenotypic and genotypic characterization. The strains were grouped into two distinct random amplification of polymorphic DNA (RAPD) biotypes harboring a range of pathogenic genes; *exb*B, *omp*H, *ptf*A, *nan*B, *sod*C, and *hgb*A. In this study, 90.90% and 81.82% *P. multocida* strains were multidrug-resistant and biofilm formers, respectively. Whole-genome sequencing of the two representative RAPD phylotypes confirmed as *P. multocida* type B: L2:ST122, harboring a number of virulence factors-associated genes (VFGs), and antimicrobial resistance (AMR) genes (ARGs). In addition, pan-genome analysis revealed 90 unique genes in the genomes of *P. multocida* predicted to be associated with versatile metabolic functions, pathogenicity, virulence, and AMR.

**Conclusion:**

This is first-ever report on the association of *P. multocida* genotype B: L2:ST122 and related VFGs and ARGs in the pathogenesis of FC in laying hens. This study also provides a genetic context for future researches on the evolutionary diversity of *P. multocida* strains and their host adaptation.

## Introduction

Poultry rearing is one of the important sources of income in Bangladesh. This subsector of livestock has been noted as the largest primary source of eggs and meat, and contributes ~3.0% to the GDP of Bangladesh [1]. However, the poultry industry in Bangladesh faces a number of constraints, including limited feed resources and frequent outbreaks of infectious diseases. Fowl cholera (FC) is an acute and fatal septicemic disease that can affect all types of birds and causes significant economic losses in poultry industries globally [2,3]. The Gram-negative bacterium *Pasteurella multocida* is the etiologic agent of FC [4]. The disease magnitudes of FC can range from acute septicemia to chronic and localized infections with wattles, sinuses, legs, wing joints, and footpads often swollen and enlarged, and mortalities have been reported up to 20% [5,6]. The route of infection is oral or nasal with transmission through nasal exudate, faces, contaminated soil, equipment, and people [7]. Different serogroups of *P. multocida* have been identified as the etiologic agent for many infectious diseases in a wide spectrum of hosts, including poultry and wild birds (FC), pigs (rhinitis and pneumonia), cattle, buffaloes, and small ruminants (hemorrhagic septicemia [HS] and enzootic pneumonia,) rabbits (snuffles), cats, dogs, and other mammals (upper respiratory tract infections and cellulitis) [8-11].

*P. multocida* is a zoonotic Gram-negative and opportunistic bacterium [12], and the strains of *P*. *multocida* are divided into five serotypes such as A (*hya*D*-hya*C), B (*bcb*D), D (*dcb*F), E (*ecb*J), and F (*fcb*D based on capsular typing [9,10]. Among these serotypes, capsular serogroups A and F cause the majority of FC, whereas serogroups B and E are predominantly associated with HS in cattle and wild ruminants [8,13]. In addition to capsular serogroups, *P. multocida* strains are currently classified into 16 Heddleston lipopolysaccharides (LPS) serovars [14,15]. Different serotypes of the *P. multocida* exhibit varying degrees of virulence in different hosts [9,10]. Various strains of *P. multocida* produce a wide arsenal of virulence factors that are crucial for pathogenesis. The virulence factor-associated genes (VFGs) include those involved in the production of a capsule, LPS, fimbriae, adhesins, and toxins, those involved in uptake and metabolism of iron and sialic acid, and those encoding hyaluronidase and certain outer membrane proteins are the key components of regulating the pathogenesis [5,8,9,16]. Although not studied extensively, many of these VFGs might play a substantial role in the pathogenesis of FC, and survival in the complex host environment [5,9,16]. The previous reports showed that there is a clear correlation between certain VFGs, and capsular types or biovars [17,18]. However, the virulence factors of *P. multocida* isolated from poultry have not been investigated in Bangladesh until now. Therefore, identification of prevalent VFGs is important to predict the pathogenic nature of the bacterium, and select potential vaccine candidates.

Control of FC is primarily ensured by disinfection management and antibacterial therapy has been used extensively in the treatment of infected individuals [6]. However, the prolonged, indiscriminated, and unnecessary overuse of antibiotics in the poultry farms have resulted in an increased incidence of antimicrobial resistance (AMR) and multi-drug resistant (MDR) isolates of *P. multocida*, posing a serious threat to public health and livestock [8]. The unplanned and irrational use of antibiotics has reduced the efficacy of most of the antimicrobial agents that are currently used in the treatment of infections in poultry infected with *P. multocida* in Bangladesh [19]. Antimicrobial susceptibility tests can provide information about the selection of appropriate antimicrobials and curtail the imprudent use of antimicrobials [20-22].

Bio-molecular techniques such as polymerase chain reaction (PCR), ribotyping, random amplification of polymorphic DNA (RAPD)-PCR, multi-locus sequence type (MLST), and *16S rRNA* gene sequencing have been used to differentiate avian strains of *P. multocida* [5,23-25]. The advantage of these methods is that all strains can be typed without depending on phenotypic properties, and the discriminatory power is generally high. In addition to these molecular techniques, whole-genome sequencing (WGS) is an affordable, convenient, and rapid technique for outbreak investigations, diagnostics, and epidemiological surveillance [26-28]. The state-of-the-art WGS technology might enable us to study the underlying genetic mechanisms associated with pathogenicity, virulence fitness, and host adaptability of *P. multocida* in multiple hosts [8,29,30]. However, there is limited information on the molecular epidemiology of *P. multocida* circulating in the global poultry industry with particular reference to Bangladesh.

Though the outbreak of FC in Bangladesh is comparatively lower compared to other countries [6] and the number of isolates is few in number, this study was aimed to utilizing different bio-molecular techniques to investigate baseline information for the characteristic analysis of *P. multocida*. In addition, we have employed the state-of-the-art WGS technology to study the genotypic diversity and underlying genetic contents such as VFGs, AMR genes (ARGs), and metabolic functional potentials of two MDR and biofilm-forming isolates of *P. multocida* causing FC in laying hens in Bangladesh. This study, for the first time, reports the diversity and genetic potentials of highly pathogenic strains of *P. multocida* type B:2 currently circulating in Bangladesh and causing FC in laying chickens of Bangladesh.

## Materials and Methods

### Ethical approval

The protocol for sampling from suspected poultry farms, sample processing, transport, and DNA extraction was approved by the Animal Experimentation Ethical Review Committee (AEERC), Faculty of Biological Sciences, University of Dhaka under Reference No. 71/Biol.Scs./2018-2019.

### Study period, sample collection, bacterial isolation, and genomic DNA extraction

A total of 57 samples (n=57) of suspected FC, including 36 live and 21 dead laying hens were collected from six commercial layer farms from August to November, 2017. These farms were located in Narsingdi (23.9193° N, 90.7176° E), Narayangonj (3.6238° N, 90.5000° E), and Manikgonj (23.8617° N, 90.0003° E) districts of Bangladesh. Diseased birds were diagnosed with FC by observing clinical signs and symptoms, including sudden death, swollen wattle and combs, lameness, respiratory rales, and diarrhea by practicing veterinarians. The birds were then dissected, and internal organs (liver) from each bird were collected as the experimental samples. The samples were finally processed, kept in the nutrient-enriched media, and transported to the laboratory (at 4°C). The collected samples were plated on Luria-Bertani broth (LB) (Oxoid, Thermo Fisher Scientific, UK). A small amount of inoculum from LB was streaked onto blood agar base (BAB) (Oxoid, Thermo Fisher Scientific, UK) supplemented with 5% sheep blood, and incubated for 24 h at 37°C for selective growth. Suspected colonies (mucoid and non-hemolytic) of the *P. multocida* on the BAB agar (3-5 colonies from each sample) were further inoculated into MacConkey agar. Colonies showing positive growth on MacConkey agar plates were further subjected to biochemical tests according to Kim *et al*. [9]. Finally, 78 (n=78) isolates of *P. multocida* were obtained through selective culture and biochemical characteristics. *P. multocida* species-specific gene (*kmt*1)-based PCR following previously published protocols [31] confirmed 22 isolates as *P. multocida*. Genomic DNA from *P. multocida* isolates was extracted from overnight culture by the boiled DNA extraction method [22]. The quality and quantity of the extracted DNA were measured using a NanoDrop ND-2000 spectrophotometer (Thermo Fisher Scientific, Waltham, MA 02451, USA). The extracted DNA was kept at −80°C until further use [32].

### Molecular typing and detection of pathogenic genes

RAPD-PCR was performed using the extracted DNA to investigate the biotype diversity of *P. multocida* in the study isolates [33,34]. The RAPD-PCR was performed using (5´-GCGATCCCCA-3´) primer following the previously optimized protocol for *Escherichia coli* [22]. Using previously published pathogenic gene-specific primers (Table-1) for PCR assays, we surveyed ten pathogenic genes in the strains of *P. multocida* [9,16]. Briefly, the PCR mixer possessed 2 mL DNA template (300 ng/mL), 10 mL PCR master mix 2X (GoTaq^®^ Colorless Master Mix, Promega, Madison, WI 53711 USA) and 1 mL (100 pmol/mL) of each primer (Table-1) in each reaction tube. The cycling condition for PCR amplifications was as follows: 94°C for 5 min; 35 cycles of 1min at 94°C, 1 min at 50-60°C, and 1 min at 72°C; and 72°C for 7 min. Amplified PCR products were visualized on 1.5% agarose gel prepared in 1× TAE buffer. After gel electrophoresis, the images were captured using Image ChemiDoc™ Imaging System (Bio-Rad, USA) [9].

**Table-1:** Primers for genotyping and pathogenic gene (virulence factors-associated genes) characterization in *Pasteurella multocida* strains.

Target gene	Gene product	Description	Primer sequence (Forward/Reverse) (5’- 3’)	Product size
*kmt*1	*Pasteurella multocida* specific	Identification of all *Pasteurella multocida* isolates	ATCCGCTATTTACCCAGTGG GCTGTAAACGAACTCGCCAC	460
*16S r*RNA	16S rRNA	P16Sf P16Sr	AGAGTTTGATYMTGGC GYTACCTTGTTACGACTT	1500
*omp*87	Outer membrane and porin proteins	Outer membrane protein 87	ATGAAAAAACTTTTAATTGCGAGC TGACTTGCGCAGTTGCATAAC	988
*tox*A	Toxin	Dermonecrotic toxin	TCTTAGATGAGCGACAAGG GAATGCCACACCTCTATAG	846
*Hsf-*1	Adhesins	Autotransporter adhesion	TTGAGTCGGCTGTAGAGTTCG ACTCTTTAGCAGTGGGGACAACCTC	664
*ptf*A	Adhesins	Fimbriae	TGTGGAATTCAGCATTTTAGTGTGTC TCATGAATTCTTATGCGCAAAATCCT GCTGG	488
*pfh*A	Adhesins	Filamentous haemagglutinin	AGCTGATCAAGTGGTGAAC TGGTACATTGGTGAATGCTG	275
*pm*HAS	Hyaluronidase	Hyaluronidase	TCAATGTTTGCGATAGTCCGTTAG TGGCGAATGATCGGTGATAGA	430
*nan*B	Neuraminidases	Neuraminidases	GTCCTATAAAGTGACGCCGA ACAGCAAAGGAAGACTGTCC	554
*hgb*A	Iron acquisition related factor	Hemoglobin binding protein	TGGCGGATAGTCATCAAG CCAAAGAACCACTACCCA	419
*exb*B	Iron acquisition related factor	Iron regulated and acquisition factors	TTGGCTTGTGATTGAACGC TGCAGGAATGGCGACTAA A	283
*sod*C	Superoxide dismutases	Superoxide dismutases	AGTTAGTAGCGGGGTTGGCA TGGTGCTGGGTGATCATCATG	235

### Antimicrobial susceptibility testing

The antimicrobial susceptibility testing of 22 isolates (*kmt*1 gene positive) of *P. multocida* was carried out using Kirby–Bauer disc diffusion method on Mueller-Hinton agar according to the guidelines of the Clinical and Laboratory Standards Institute; CLSI [35] and European Committee on Antimicrobial Susceptibility Testing; EUCAST [36]. Antibiotics were selected for susceptibility testing corresponding to a panel of antimicrobial agents of interest to the poultry industry and public health in Bangladesh. Seventeen (n=17) antibiotic discs comprising ampicillin (AMP) (10 mg), oxacillin (OXA) (10 mg), doxycycline (DOX) (30 mg), streptomycin (STR) (10 mg), nitrofurantoin (F) (300 mg), levofloxacin (LEV) (third-generation) (5 mg), aztreonam (ATM) (30 mg), cefoxitin (Fox) (30 mg), gentamicin (CN) (10 mg), nalidixic acid (NA) (first generation) (30 mg), trimethoprim (Tm) (5 mg), tetracycline (TE) (30 mg), ciprofloxacin (CIP) (second generation) (5 mg), chloramphenicol (C) (30 mg), sulfonamide (S3) (250 mg), colistin sulfate (CS) (10 mg), and cefepime (FEP) (30 mg) were used for antimicrobial susceptibility testing. The susceptibility pattern of *P. multocida* isolates was categorized as susceptible, intermediate, and resistant according to the CLSI and EUCAST breakpoints.

### Biofilm formation (BF) assay

The BF ability of the *P. multocida* isolates (n=22, *kmt*1 gene positive) was tested in 24-well polystyrene plates (Corning, Costar) following previously published protocols [37]. Briefly, the *P. multocida* isolates were revived in LB medium for 24 h at 37°C with shaking. A 1:1000 dilution of the LB was prepared and 25 mL of diluted LB was placed in each well containing 1.5 mL of culture medium. The plates were incubated for 48 h at 37°C in static condition. Planktonic cells were removed, and wells containing biofilms were rinsed 3 times with distilled water. Finally, the remaining adherent bacteria in the wells were stained with 2 mL/well of crystal violet (CV) (0.7% [wt/vol] solution; Sigma-Aldrich) for 12 min. The excess stain was removed by washing with distilled water. CV was extracted by acetic acid (33% [vol/vol]), and the plates were incubated at room temperature to release the dye into the solution. Then, 100 mL samples from each well were transferred to a 96-well flat-bottom plate, and the amount of dye was determined at 600 nm using a microplate reader [20]. The solution was removed, and the absorbance was measured at optical density-590 (OD590), and this step was repeated 3-times. To determine the BF ability of strains, cutoff OD (ODc) was defined as three standard deviations above the mean OD of the negative control. Strains were classified as: Non-biofilm formers, (NBF) (OD ≤ODc); weak biofilm formers, (WBF) (ODc <OD ≤2 ×ODc); moderate biofilm formers, (MBF) (2 ×ODc <OD ≤4 ×ODc), and strong biofilm formers, (SBF) (OD >4 ×ODc) [37]. In this study, the ODc value was set as 0.041, and the mean OD of the negative control was 0.035±0.012 [37]. The biofilm surfaces were then visualized using 5% TSB as nutrient-rich media, and FilmTracerTM LIVE/DEAD^®^ Biofilm Viability Kit (Thermo Fisher Scientific, Waltham, MA 02451, USA) as staining materials to observe the proportion of live or active cells (fluorescent green) under Olympus BX51 upright microscope (40× objective). Finally, images were captured using an Olympus DP73 camera through cellSens entry software (Olympus Corporation, Japan) and visualized using Java-based image processing program ImageJ developed at the National Institutes of Health and the Laboratory for Optical and Computational Instrumentation, USA [20].

### Ribosomal gene (16S rRNA) sequencing and phylogenetic analysis

Two *P. multocida* isolates; PM4 and PM7, representative of each genotype and/or biotype, were selected for 16S rRNA sequencing using universal primers (Table-1). WGS was carried out at the First Base Laboratories SdnBhd (Malaysia) using BigDye^®^ Terminator v3.1 cycle sequencing kit (Thermo Fisher Scientific, Waltham, MA 02451, USA) chemistry [37] under Applied Biosystems highest capacity-based genetic analyzer (ABI PRISM^®^ 377 DNA Sequencer, Applied Biosystems, USA) platform. Initial quality control of the generated raw sequences was performed using SeqMan software (DNASTAR, Inc.3801 Regent St. Madison, USA), and Molecular Evolutionary Genetics Analysis (MEGA) version 7.0 (Institute of Molecular Evolutionary Genetics, Pennsylvania State University, University Park, USA) for bigger datasets [38] was used to align the WGS with relevant reference sequences retrieved from NCBI database. A phylogenetic tree of *16S rRNA* genes was reconstructed using the neighbor-joining method [32] implemented in the MEGA 6.0 software with the Kimura-Nei method [39]. Bootstrap values were calculated with 1000 resamples.

### WGS and assembly

Two RAPD representative *P. multocida* pathotypes (PM4 and PM7) were selected for WGS. Genomic DNA libraries containing 300~500 bp fragments were constructed using Nextera XT DNA Library Preparation Kit (Illumina Inc., San Diego, USA). The WGS was performed using 100 bp paired-end sequencing protocol under Illumina platform using HiSeq4000 sequencer (Macrogen, lnc. Seoul, Republic of Korea) with an average ~456-fold genome coverage per sample. The generated FASTQ files were evaluated for quality using FastQC v0.11 [40]. Adapter sequences, and low-quality ends per reading were trimmed by using Trimmomatic v0.39 [41] with set criteria of sliding window size 4; a minimum average quality score of 20; and minimum read length of 50 bp. High-quality reads were undergone to *de novo* assembly using SPAdes (Species Prediction and Diversity Estimation) v3.13 [42] with a range of k-mer between 21 and 121. Once assembled, the contigs for each bacterial sample were screened and those contigs that were <500 bps, possessed low coverage scores, and/or were poorly associated with *P. multocida* species were removed from the assemblies. The draft contigs were mapped, reordered, and scaffolded according to an NCBI reference complete sequence of *P. multocida* strain Razi_Pm0001 (accession number: NZ_CP017961.1) by RaGOO v1.1 [43]. The completeness of the scaffold was checked using CheckM [44]. Scaffolded contigs were searched for the bacterium at strain level by BLAST, and the k-mer algorithm in the KmerFinder 3.1 tool [45]. The WGS-based phylogenetic tree was constructed using the online pipeline Reference Sequence Alignment Based Phylogeny Builder (REALPHY) [46], and visualized on iTor v5 [47]. The Plasmid Finder v2.1 and Integron_Finder v1.5 tools were used for the detection of plasmid sequence contamination [48,49]. Prophage sequences within the genome assemblies were identified by the PHAge Search Tool Enhanced Release (PHASTER) webserver [50].

### Genome annotation and genomic organization mapping

The scaffolded genomes of the *P. multocida* PM4 and PM7 strains were annotated by multiple annotation schemes to improve accuracy. We used the NCBI Prokaryotic Genome Annotation Pipeline (PGAPv4.11) with best-placed reference protein set, and GeneMarkS-2+ annotation methods [51], Rapid Prokaryotic Genome Annotation (Prokka) (e=0.000001) [52], and Rapid Annotation using Subsystem Technology (e=0.000001) [53]. Annotated genes by each tool were then cross-checked. To remove the tRNA and mRNA from the genomes, we used tRNAscan-SE v2.0 [54], and Aragorn v1.2.38 [55] tools. The graphical map of the circular genome was generated using the CGView Server (http://stothard.afns.ualberta.ca/cgview_server/) [56]. Circular BLAST of annotated genome comparison was performed using an in-house instance of BLAST Ring Image Generator v0.95 [57]. The pan-genome and core-genome were analyzed, mapping the genomes against the reference genomes of *P. multocida* strains from avian and type B from bovine species (with at least 90% DNA identity and 90% genome coverage) using the Bacterial Pan Genome Analysis pipeline [58].

### Genome sequence analysis

The assembled genomes of PM4 and PM7 were aligned with *P. multocida* capsular serotypes (*hya*D*-hya*C*, bcb*D*, dcb*F*, ecb*J, and *fcb*D) [9]; LPS genotypes (LPS outer core structure genes) [15] genes using Burrows-Wheeler Aligner (BWA) [59]. The output was extracted using Resistome Analyzer integrated into AmrPlusPlus v2.0 pipeline [60]. MLST genotypes of the *P. multocida* strains were assigned by performing BLAST of their scaffolds against the *P. multocida* MLST Databases (https://pubmlst.org/pmultocida). In this study, RIRDC MLST, a scheme of multi-locus sequence typing based on seven housekeeping genes (*adk, est, pmi, zwf, mdh, gdh*, and *pgi*) of *P. multocida* in the Bacterial Isolate Genome Sequence Database (BIGSdb) [61] was used to determine the MLST genotypes of the isolates (PM4 and PM7).

### Virulence factors gene (VFG), ARG and metabolic functions profiling

The genomes of PM4 and PM7 were further aligned with *P. multocida* outer membrane and porin proteins (*oma*87*, omp*H*, plp*B, and *psl*), adesins (*ptf*A*, fim*A*, hsf-1, hsf-2, pfh*A*, tad*D); neuraminidases (*nan*B and *nan*H), iron acquisition related factors (*exb*D*, ton*B*, fur, tbp*A*, hgb*A, and *hgb*B); superoxide dismutases (*sod*A and *sod*C*);* dermonecrotoxin *(tox*A); and hyaluronidase (*pm*HAS) pathogenic genes using BWA [59]. The output was extracted using Resistome Analyzer integrated into AmrPlusPlus v2.0 pipeline [60]. We further mapped the annotated genomes against the ResFinder Database [62], Comprehensive Antibiotic Resistance Database (CARD) [63], MEGARes [60,64] databases to search for ARGs. The gene fraction value ≥95 was considered for ARGs to our study strains. The generated assemblies for each of the *P. multocida* strains were analyzed by translated BLAST against a dataset of *P. multocida* virulence factors extracted from the virulence factor database using an e-value cutoff of 10^−5^ [65] for putative VFGs identification. The metabolic functional potentials of the genomes were predicted through the KEGG (Kyoto Encyclopedia of Genes and Genomes) Automatic Annotation Server (KAAS) [66] database.

## Results and Discussion

The FC is a highly contagious bacterial disease of domestic and wild birds and is considered as a major threat to the poultry industries worldwide [6,7,67]. The disease is often associated with severe economic loss in the poultry industry due to high mortality and production loss. Vaccination is one of the most important strategies for the prevention and control of FC in poultry farms [67]. In Bangladesh, there are just a few research papers on the isolation and identification of *P. multocida* from commercial poultry farms [67-69]. However, as far we know, none of these studies were carried out in detail at the molecular level using advanced biomolecular and genome sequencing techniques. Therefore, following a need to analyze the diversity and genetic potentials of highly pathogenic strains of *P. multocida*, we employed different molecular genetic techniques, *in vitro* resistance (antibiogram and BF) assays, and ribosomal gene sequencing. In addition, we performed WGS of two MDR and biofilm-forming *P. multocida* isolates to detect the VFGs, ARGs, and metabolic functional potentials related to the pathophysiology of FC.

A total of 78 *P*. *multocida* isolates were obtained from 57 samples originating from different layer farms of Bangladesh based on their distinct cultural characteristics, non-motile and non-hemolytic phenomena (Table-S1). Of the retrieved isolates (n=78), the species and type-specific PCR using *kmt*1 gene confirmed 22 isolates as *P*. *multocida* (Table-2, Figure-S1), which were further confirmed through biochemical and molecular characterization [9,31]. The *kmt*1 gene-positive 22 isolates of *P. multocida* were catalase and oxidase-positive, and urease-negative. These isolates did not show any reaction in citrate, methyl red, and Voges-Proskauer tests (Table-2). However, they fermented glucose, mannose and sucrose, and none utilized lactose. In addition, inositol fermentation grouped these isolates into two biotypes such as 14 (63.64%) isolates fermented inositol, and denoted as *P. multocida* biotype 1, and 8 (36.36%) isolates were unable to ferment inositol, and thus denoted as *P. multocida* biotype 2 (Table-2). These results indicated that *P. multocida* might possess diverse metabolic potentials despite being identified from the same outbreak of FC, corroborating previous findings [70]. In this study, the RAPD profiling indicated less genetic heterogeneity among the studied *P. multocida* strains and confirmed two pathotypes of *P. multocida* among these 22 strains (Table-2, Figure-S2). Of the detected pathotypes, 14 isolates of *P. multocida* biotype 1 showed the RAPD pattern 1 and eight isolates of *P. multocida* biotype 2 showed RAPD pattern 2 (Table-2). These findings are in line with several previous reports on RAPD biotyping of *P. multocida* [71]. Moreover, pathogenic gene-specific primer-based PCR identified different pathogenic genes among the identified *P. multocida* strains (Table-2). Three VFGs, namely, *nan*B, *sod*C, and *hgb*A were found in all of the 22 isolates, while 22.73% (5/22) isolates harbored six VFGs (*exb*B, *omp*H, *ptf*A, *nan*B, *sod*C, and *hgb*A) (Table-2). The findings of this study demonstrated ubiquitous presence of VFGs in *P. multocida*, indicating their high pathogenic potentials to causing FC. The pathogenicity of *P. multocida* is reported to be associated with various virulence factors and the wide distribution of VAGs is significant for the survival of *P. multocida* in the host environment [7,9,70]. In addition, the biochemical and molecular findings of the present study corroborated with the previous findings of Omaleki *et al*. [30].

**Table-2:** The biomolecular features, and virulence factors-associated genes profile of the *Pasteurella multocida* isolates (n=22).

Isolates	Biotype	*kmt1*	Pathogenic genes
PM1	1	+	*exb*B*, omp8*7*, hgb*A
PM2	1	+	*exb*B*, omp8*7*, ptf*A
PM4	2	+	*exb*B*, omp8*7*, ptf*A*, nan*B*, sod*C*, hgb*A
PM5	1	+	*omp*87*, ptf*A*, nan*B*, sod*C*, hgb*A
PM7	1	+	*exb*B*, omp*87*, ptf*A*, nan*B*, sod*C*, hgb*A
PM10	2	+	*exb*B*, sod*C*, hgb*A
PM11	1	+	*exb*B*, omp*87*, ptf*A*, nan*B
PM12	2	+	*nan*B*, sod*C*, hgb*A
PM13	1	+	*nan*B*, sod*C*, hgb*A
PM14	1	+	*exb*B*, omp*87*, ptf*A*, nan*B*, sod*C
PM15	2	+	*exb*B*, omp*87*, ptf*A*, nan*B*, sod*C*, hgb*A
PM19	2	+	*exb*B*, omp*87*, ptf*A*, nan*B*, sodC, hgb*A
PM21	2	+	*exb*B*, omp*87*, ptf*A*, nan*B*, sod*C*, hgb*A
PM22	1	+	*nan*B*, sod*C*, hgb*A
PM26	1	+	*nan*B*, sodC, hgb*A
PM30	1	+	*nan*B*, sod*C*, hgb*A
PM31	2	+	*exb*B*, omp*87*, ptf*A*, nan*B*, sod*C*, hgb*A
PM34	1	+	*nan*B*, sod*C*, hgb*A
PM36	1	+	*exb*B*, omp*87*, sod*C*, hgb*A
PM39	2	+	*nan*B*, sod*C*, hgb*A
PM43	1	+	*nan*B*, sod*C*, hgb*A
PM44	1	+	*nan*B*, sod*C*, hgb*A

“+”=presence; Biotype: 1(Glucose+, Inositol+, Lactose -, Mannitol +, Mannose +, Sucrose +, Dulcitol -, Xylose +, Indole production +, MR-VP -, Urease -, H2S production -, Citrate utilization -, Catalase +, Oxidase +), Biotype: 2 (Glucose+, Inositol-, Lactose -, Mannitol +, Mannose +, Sucrose +, Dulcitol -, Xylose +, Indole production +, MR-VP -, Urease -, H2S production -, Citrate utilization -, Catalase +, Oxidase +) represent two different RAPD patterns

**Figure-1:**
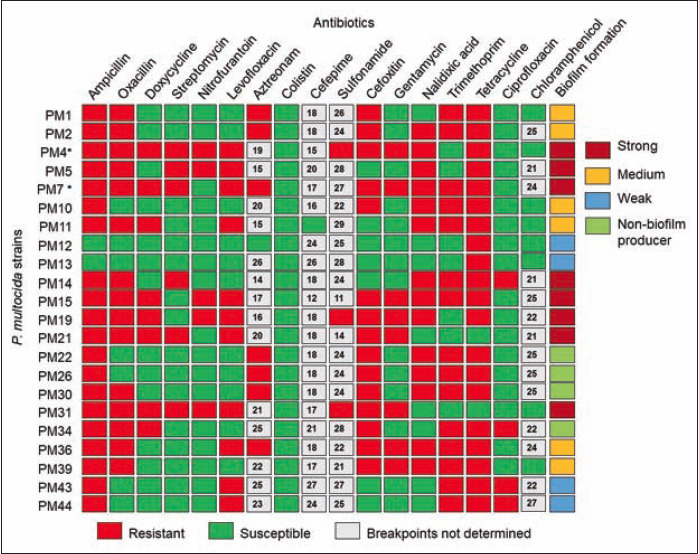
Antimicrobial resistance profile of the tested 22 *Pasteurella multocida* strains. Antibiotic susceptibility to 17 antibiotics of varied classes was determined by disk diffusion assays. The strains were categorized as resistant or susceptible based on the breakpoints defined by the European Committee on Antimicrobial Susceptibility Testing (EUCAST, 2020). Numeric inside the gray boxes represent zone of inhibition (in mm unit) for the antibiotics that have no standard breakpoints currently available. Biofilm producing abilities are shown in the right column based on their adherence potential on 24-well polystyrene plates. The superscript asterisks (*) in two isolates (PM4 and PM7) indicate that they were selected for whole-genome sequencing.

Antibiotic therapy is still considered as a tool in the treatment of FC. However, AMR has become a global problem as resistant isolates have emerged by the excessive and unjustified use of antimicrobials [5,19,67]. The *P. multocida* strains of the present study showed resistance to ampicillin (90.91%), tetracycline (90.91%), and nalidixic acid (63.64%) according to the EUCAST breakpoints (Figure-1). In addition, 68.18% *P. multocida* strains were found to be resistant against each of the three classes of antibiotics (e.g., oxacillin, cefoxitin, and trimethoprim) tested. Remarkably, 91.91% *P. multocida* strains were multidrug-resistant (resistant against ≥5 antibiotics) (Figure-1). The results on the AMR of the present study, consistent with previous AMR reports from across the globe, including Malaysia [25], France [72], Japan [73], and China [74] indicated that ampicillin, tetracycline, and nalidixic acid were the most resistant antibiotics. However, all of the tested isolates were sensitive to colistin, suggesting that colistin could effectively be used for the treatment of FC with *P. multocida* considering the serious worldwide concern on AMR. Frequent and excessive use of antibiotics in the livestock of Bangladesh [19,21] might have a role in AMR development against multiple antibiotics in clinical infections like FC [75,76].

**Figure-2:**
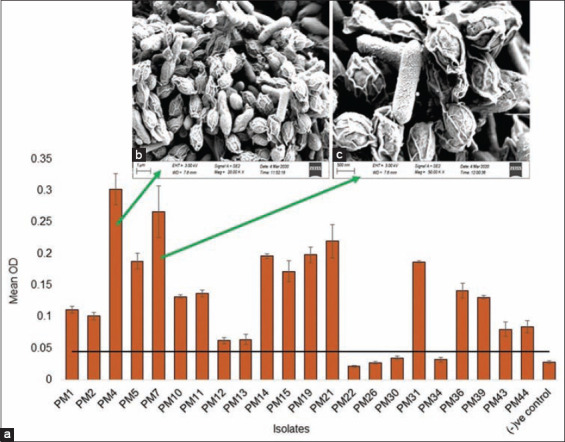
Biofilm formation ability of the Pasteurella multocida isolates. (a) Mean optical density of the 22 isolates measured at 600 nm after 48 h growth at 37°C. The horizontal line represents the threshold below which indicates non-biofilm producers. Biofilms of two strong biofilm-producing isolates, (b) PM4 and (c) PM7 were further observed under scanning acoustic microscopy (SAM). The SAM micrographs demonstrated the colonizing bacterial cells after 48 h incubation, and revealed how the bacteria tend to grow in clumps (micro-colonies), and the exopolysaccharide that is covering the bacteria. Error bars represent standard deviation.

The formation of biofilms and subsequent encasement of bacterial cells in a complex matrix can enhance resistance to antimicrobials and subsequently making *P. multocida* difficult to eradicate and control [20]. In this study, 81.82% *P. multocida* strains were biofilm-formers with significant differences (p=0.039) in their BF categories. Of the tested isolates, 36.37%, 27.27%, and 18.18% were SBF, MBF, and WBF, respectively (Figure-2a). The development of bacterial biofilms is presently recognized as one of the most relevant drivers of infections and one of the reasons for treatment failure with antibiotics [76-78]. In our present study, 18.18% of study strains were found as NBF (Figure-2a). In addition, the SBF strains of *P. multocida* showed higher AMR properties (41.18-64.71%) compared to the MBF (14.50-34.65%), and WBF (5.88-29.91%) strains. Moreover, 35.0-41.0% of the NBF strains also showed AMR phenomena against these antibiotics (Figure-1). Scanning acoustic microscopy of the two representatives *P. multocida* strains (Isolate: PM4 and PM7), as representative of SBF strains demonstrated colonization densely and exopolysaccharides covering the bacterial cells, validating the high biofilm potential of these two MDR strains (Figures-2b and c). Therefore, results of the antibiogram and BF assays indicated that BF ability of the *P. multocida* may enhance antibiotic resistance and pathogenic fitness to survive under unfavorable complex conditions within host and environmental niches [76]. Moreover, the biofilms may also promote the bacterium to resist host immune defense mechanisms [76].

**Figure-3:**
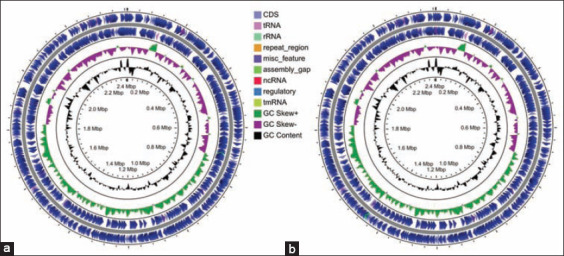
Circular representation of the genome of of *Pasteurella multocida* (a) PM4 and (b) PM7 strains. The PM4 and PM7 genomes, and their coding regions with homologies, the tRNA and rRNA operons, and the overall G-C content are presented. The outer two circles demonstrate the coding sequence, tRNA, and rRNA. The third circle shows the GC content (black). The fourth circle represents the GC skew curve (positive GC skew, green; negative GC skew, violet). The figures were generated by using CGView Server (http://stothard.afns.ualberta.ca/cgview_server/).

**Figure-4:**
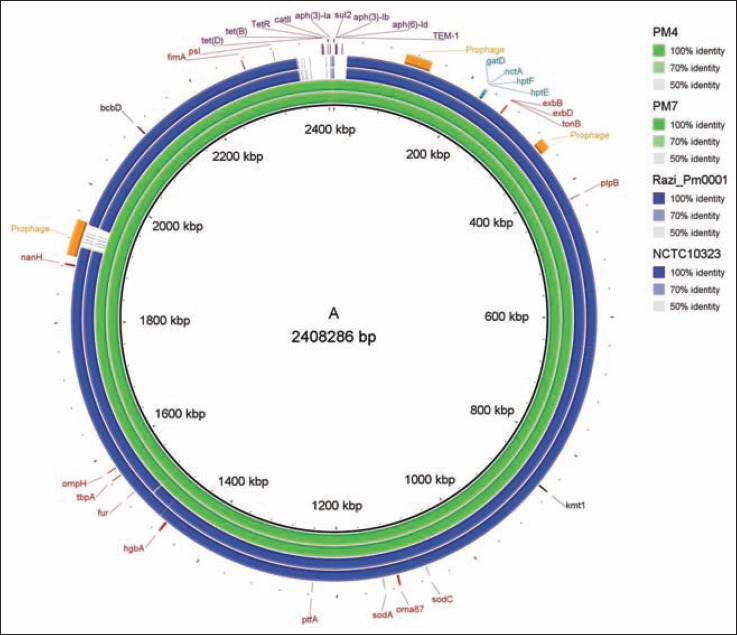
Distribution of virulence factors-associated genes (VFGs), antibiotic resistance genes (ARGs), and prophage related sequence features. Genomes of *Pasteurella multocida* strains Razi_Pm0001 and NCTC10323 found to be more closely related to *P. multocida* PM4 and PM7 strains. Capsular serotype determining gene (black), lipopolysaccharide genotyping genes (teal), VFGs (red), ARGs (purple), and prophage (orange) are presented with their respective genomic positions.

Two strains of *P. multocida*, initially identified through ribosomal gene (*16S rRNA*) sequencing, biotyping, and RAPD grouping with MDR and SBF phenomena, were subjected to WGS. The *16S rRNA* gene-based phylogenetic analysis revealed that these two *P. multocida* strains had 99.9% identity to Razi_Pm0001 (GenBank accession number: NZ_CP017961.1), and clustered with previously identified *P. multocida* strains (Figure-S3). The previous investigations have also shown that *P. multocida* associated with FC represented multiple clones [8,79]. We found 34 and 37 contigs in PM4 and PM7 strains of *P. multocida*, and the average genome length of these two strains were 2,408,286 bp and 2,408,436 bp, respectively (Figure-3, Table-3). The average GC content of each genome was 40.4% (Table-3), which was consistent with that of a complete *P. multocida* chromosome [10,14]. The genome completeness of both strains was 99.55% with a genome coverage 458× and 455.0× for PM4 and PM7, respectively (Figure-3, Table-3). The genome assembly and annotations statistics of the PM4 and PM7 complete genomes are summarized in Table-4. The PM4 and PM7 genomes contained 2260 and 2261 coding sequences, respectively, where 2217 and 2221 protein-coding genes were, respectively, found in these genes. Moreover, the number of RNA genes was 54, which included 50 transfer RNAs (tRNAs) and 4 rRNAs in each genome (Table-4, Figure-3). In addition, three intact prophages were found in both genomes compared to the two intact and one incomplete prophage within the reference strain Razi_Pm0001 (Figure-4). Despite differences in biotype and RAPD profiles (Table-2, Figure-S2), both of the strains showed similar genomic features (Table-3). The PM7 strain harbored two unique genes, and of them, one encoding tonB-dependent hemoglobin/transferrin/lactoferrin family outer membrane receptor facilitating the use of transferrin, lactoferrin, and hemoglobin as sources of iron in different hosts as also reported previously [80]. Strikingly, endoU domain-containing protein and inositol-1-monophosphatase genes were found in both PM4 and PM7 *P. multocia* strains with 100% amino acid sequence identity, and these genes/proteins are involved in inositol metabolism. The diversity of inositol fermentation by PM4 (inositol −) and PM7 (inositol +) possibly related to the expression of the gene, which needs further investigations. These findings indicated all biotypic and RAPD clustering of *P. multocida* does not necessarily related to genotypic clusters rather expression of genes plays a determining role in phenotypic classification [81,82].

**Table-3:** Summary of genome assembly and genotypic characteristics of whole-genome sequenced PM4 and PM7 strains of *Pasteurella multocida*.

Strains	Host origin	Clinical syndrome	Capsular genotypes	Lipopoly saccharide genotypes	Multi-locus sequence type genotype	No. of Contigs	No. of Contigs after scaffolding	GC (%)	Coverage	Size (bp)	Completeness
PM4	chicken	Fowl cholera	B	L2	ST122	34	1	40.4	458×	2,408,286	99.55
PM7	chicken	Fowl cholera	B	L2	ST122	37	1	40.4	455×	2,408,436	99.55

**Table 4:** Assembly and annotation statistics of the PM4 and PM7 strains of *Pasteurella multocida.*

Features	PM4	PM7
**NCBI Prokaryotic Genome Annotation Pipeline**		
(GeneMarkS-2+)		
Genes (total)	2.318	2.319
CDSs (total)	2.260	2.261
Genes (coding)	2.217	2.221
Genes (RNA)	58	58
Complete rRNAs (5S, 16S, 23S)	2, 1, 1	2, 1, 1
tRNAs	50	50
ncRNAs	4	4
Pseudo Genes (total)	43	40
**RASTtk v2.0**		
Number of Coding Sequences (CDS)	2.323	2.332
Gene in subsystem		
Non-hypothetical	814	817
Hypothetical	39	39
Gene not in subsystem		
Non-hypothetical	992	993
Hypothetical	478	483
Number of RNAs	56	56
**Prokka v1.12**		
Number of genes predicted	2.313	2.313
Number of protein-coding genes	2.258	2.258
Number of genes with non-hypothetical function	1.684	1.682
Number of genes with EC-number	890	890
Number of genes with seed subsystem ontology	745	745
**tRNAscan-SE v. 2.0**		
Total tRNAs	52	52
**ARAGORN v1.2.38**		
Total tRNA genes	50	50
Total tmRNA	1	1

**Figure-5:**
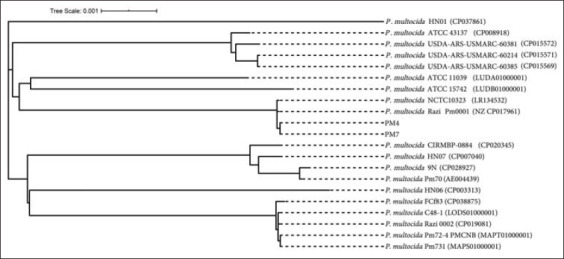
Complete genome-based phylogenetic analysis of *Pasteurella multocida* strains. The phylogenetic tree was constructed using the Reference Sequence Alignment Based Phylogeny Builder (REALPHY).

The WGS-based phylogenetic tree revealed that *P. multocida* PM4 and PM7 strains clustered in the same branch with the serotype B strains Razi_Pm0001 and NCTC10323 of NCBI of the reference genomes (Figure-5). The presence of *bcb*D gene in the genomes of PM4 and PM7 determined the capsular serotype B for both strains. The gene *bcb*D is associated with capsular biosynthesis of *P. multocida* specific to serogroup B [9,83]. Conversely, rest of the *P. multocida* isolates of the present study showed a higher degree of similarity in RAPD pattern (Figure-S2), suggesting that these isolates likely to be belonged to the same genotype. These findings are in line with Hotchkiss *et al*. [71] who reported that closely related *P. multocida* isolates show a similar RAPD pattern. The LPS outer core structural genes *gat*D, *nct*A, *hpt*F, and *hpt*E found in both the isolates indicating LPS genotype L2 as also reported in several earlier studies [9,15]. Furthermore, the seven housekeeping genes (*adk*, *est*, *pmi*, *zwf*, *mdh*, *gdh*, and *pgi*) of *P. multocida* assigned both isolates into ST122 which is widely documented to be associated with bovine HS (Table-3, Figure-4). Notably, *P. multocida* from genotype B: L2:ST122 is predominant in bovine [8], and no previous reports show the association of this genotype FC in avian species. Moreover, majority of the avian pasteurellosis outbreaks are caused by *P. multocida* from types A and D [8,15]. However, cross-species transmission of diseases is frequently reported by different serotypes of *P. multocida*, that is, serogroups A and D. These serotypes are globally distributed and found to cause diseases in a wide range of domestic animals (e.g., from fowl to calves, pigs, sheep, goats, and rabbits) [13,15,70]. Conversely, *P. multocida* serogroups B and E have been found predominantly in tropic areas where they induce HS in cattle and wild ruminants [13,15,70]. Therefore, it is assumed that *P. multocida* type B: L2:ST122 infections manifesting FC in commercial layer birds might have occurred through host adaptation and spill-over transmission of the strains from bovine into chicken. A mixed cultivation system of cattle, goat, sheep, and poultry together in the rural areas of Bangladesh as well as poor hygienic practices in the farms further increase the possibility of cross-species transmission [84].

The genomic features such as coverage, identity, and product of different VFGs identified in the complete genomes of the two study strains are shown in Table-5. In this study, we found different VFGs encoding for outer membrane and porin proteins (*oma*87, *omp*H, *plp*B, and *psl*), adhesins (*ptf*A and *fim*A), neuraminidases (*nan*H), iron acquisition related factors (*exb*B, *exb*D, *ton*B, *fur*, *tbp*A, and *hgb*A), and superoxide dismutases (*sod*A and *sod*C) in the assembled genome of both PM4 and PM7 isolates (Figure-4). This result is in agreement with the PCR-based virulence profile of the *P. multocida* strains studied (Table-1, Figure-4). Fourteen potential VFGs were identified in the PM4 and PM7 genomes with 100% query coverage (Table-5). These gene-products are involved in many biosynthetic pathways, including secretion system and its effectors, several phospholipases, the elastase and protease IV enzymes, production of phenazines, the exotoxin-A, quorum sensing systems, and synthesis and uptake of the pyochelin siderophore that might have an important role in survival and pathogenesis of *P. multocida* strains avian species [8,15]. The high frequency of VFGs analyzed was also observed in other studies with strains from both avian and animal hosts [8,14,15,83]. A comprehensive search for ARGs in the complete genomes of *P. multocida* (PM4 and PM7 genomes) explored an array of ARGs (Figure-4, Table-6). Both PM4 and PM7 genomes seem to be well equipped with a similar load of drug resistance genes conferring resistance to aminoglycoside [*aph*(3’)-Ia, *aph*(3’’)-Ib, *aph*(6)-Id], sulfonamides (*sul*2), tetracyclines (*tet*A, *tet*D), phenicol (*cat*II), trimethoprim (*dfr*), and b-lactam (*TEM*-1). The ARGs were categorized into efflux pump conferring antibiotic resistance, antibiotic inactivation enzyme, and antibiotic target in susceptible species (Table-6). The presence of ARGs in PM4 and PM7 genomes corroborated with the resistance pattern observed in antibiotic susceptibility tests (Figure-1, Table-6). Like other Gram-negative bacteria, tetracycline-resistant *P. multocida* frequently possesses genes *tet*D and *tet*A, implicating resistance to tetracycline through efflux of tetracycline or for a protein that prevents tetracycline binding to the bacterial ribosome [25,85]. Both of the study strains were resistant to tetracycline, ampicillin, nalidixic acid, oxacillin, cefoxitin, and trimethoprim according to EUCAST breakpoints. The zone of inhibition (ZOI) for PM7 strain was 27 mm (Figure-1). In addition, all of the study isolates, including PM4 and PM7 were sensitive to colistin corroborating with the absence of *pmr*E gene in their genomes or cannot play a role in LPS modification to conferring resistance [25]. The *pmr*E gene leads to the production of l-Ara4N and pEtN, both of which are responsible for the acquisition of colistin resistance [28]. Remarkably, we did not find any plasmid and integrin representing sequences in the genomes of PM4 or PM7 indicating the chromosomal origin of the ARGs [8].

**Table-5:** Genome-wide distribution of virulence factors-associated genes, and their features in PM4, PM7 and Razi_Pm0001 strains.

Gene	Query Coverage (%)	Query identity (%)	Product
*lpx*C	100	91	UDP-3-O-[3-hydroxymyristoyl] N-acetylglucosamine deacetylase
*rfa*D	100	90	ADP-L-glycero-D-manno-heptose-6-epimerase
*gal*E	100	86	UDP-glucose 4-epimerase
*rfa*E	100	85	D-glycero-beta-D-manno-heptose 1-phosphate adenylyltransferase/D-glycero-beta-D-manno-heptose-7-phosphate kinase
*yhx*B*/man*B	98	88	Phosphoglucosamine mutase
*wec*A	98	83	Undecaprenyl-phosphate alpha-N-acetylglucosaminyl 1-phosphate transferase
*msb*A	98	80	Lipid A export permease/ATP-binding protein MsbA
*orf*M	97	83	Nucleoside 5-triphosphatase RdgB (dHAPTP, dITP, XTP-specific)
*gal*U	96	87	UTP--glucose-1-phosphate uridylyltransferase
*lpx*B	96	81	Lipid-A-disaccharide synthase
*kds*A	94	92	2-Keto-3-deoxy-D-manno-octulosonate-8-phosphate synthase
*gmh*A*/lpc*A	94	91	D-sedoheptulose 7-phosphate isomerase
*rfa*F	91	83	ADP-heptose--lipooligosaccharide heptosyltransferase II
*msb*B	80	80	Lipid A biosynthesis myristoyltransferase

**Table-6:** Antimicrobial resistance genes in the genomes of PM4 and PM7 strain.

Gene	Product	Resistance mechanism
*aph* (3’’)-Ib	Aminoglycoside 3’’-phosphotransferase	Antibiotic inactivation enzyme
*sul*2	Dihydropteroate synthase type-2, Sulfonamide resistance protein	
*cat*II	Chloramphenicol O-acetyltransferase	
*TEM*-1	Class A beta-lactamase	
*aph (3’)-Ia*	Aminoglycoside 3’-phosphotransferase	
*aph (6)-*Id	Aminoglycoside 6-phosphotransferase	
*tet*D	Right origin-binding protein	Efflux pump contributing resistance
*tet*A	Tetracycline resistance, MFS efflux pump	
*tet*R	Tetracycline resistance regulatory protein TetR	
*TUF*AB	EF-Tu inhibition protein	Antibiotic target in susceptible species
*fol*A*, dfr*	Dihydrofolate reductase	
*rho*	Transcription termination factor Rho	

**Figure-6:**
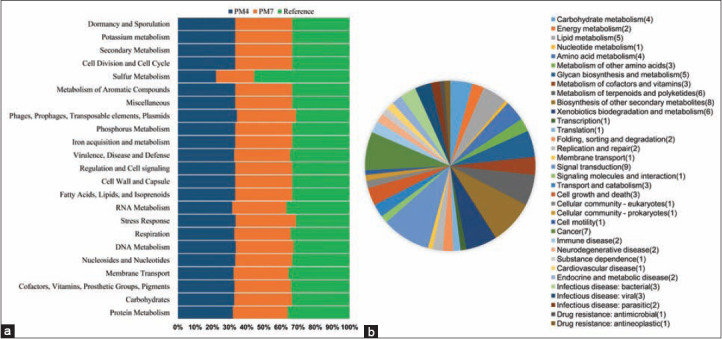
Metabolic pathway reconstruction and subsystem distribution. (a) Comparative gene distribution to different metabolic pathways of PM4, PM7, and the closely related Razi_Pm0001 genomes as predicted by Rapid Annotation System Technology Server. (b) The genes classified into 34 categories, and 356 subsystems. The pie chart and count of the subsystem features in the right panel demonstrate the percentage distribution and category of the subsystems found in all the three strains PM4, PM7, and Razi_Pm0001, predicted using KEGG pathway analysis.

The metabolic functional potential annotations of the PM4 and PM7 genomes of *P. multocida* through KEGG pathways classified the genes into 34 categories, and 356 subsystems (Figure-6). In these genomes (PM4 and PM7), 37% of genes were found to be coding for different subsystems, and rest of the genes were out of the subsystem list. Conversely, RASTtk [86] annotation identified genes related to cellular process, metabolism, environmental information processing, genetic information processing, and pathogenesis (Figure-6a). Of the predicted subsystems, “amino acids and derivatives” was the largest functional pathway accounting for 190 genes in PM4 and PM7, while the reference strain (Razi_Pm001) harbored 199 genes. Likewise, functional pathways related to “protein metabolism” identified 175 genes in PM4 and PM7 genomes, and 197 in the reference strain (Razi_Pm001). The genome of PM4 harbored 134 genes coding for metabolism of “carbohydrates” whereas 142 and 141 genes were, respectively, found in the genomes of PM7 and reference strain. In addition, 116 genes associated with “cofactors, vitamins, prosthetic groups, and pigments” metabolism were identified in the study genomes (PM4 and PM7) while the reference strain had 120 genes to be related to this metabolic functional pathway (Figure-6a). These pathways include flagellar biosynthesis, motility, quorum sensing, BF, biosynthesis of vitamin, co-factors, folate, xenobiotics metabolism, and so on (Figure-6b). Diverse metabolic pathways of the bacterial strains reflect their pathogenic fitness, and robust to cause multiple diseases in different hosts [78].

**Figure-7:**
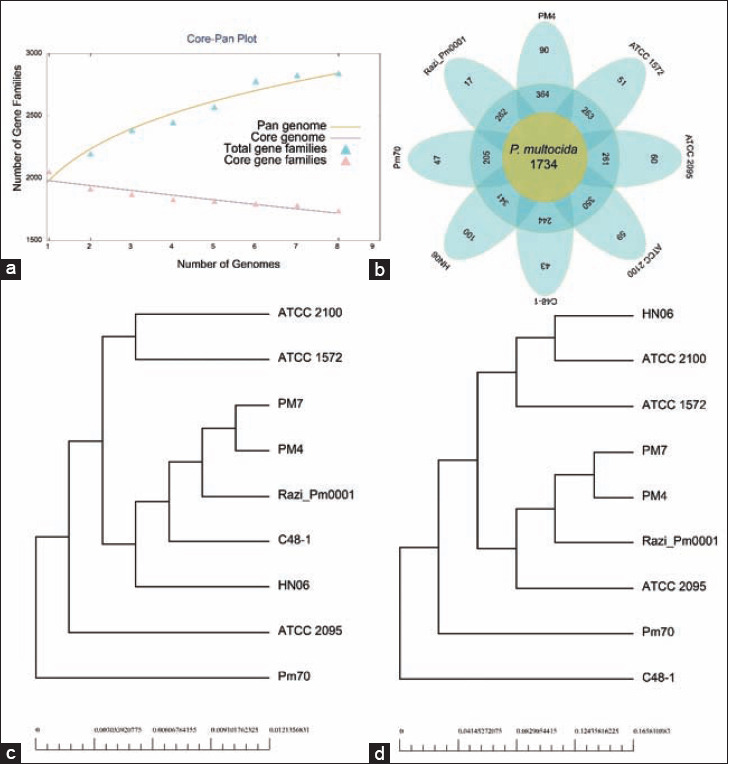
Pan-genome analysis of seven *Pasteurella multocida* strains in the repertoire of GenBank. (a) Pan-genome and core genome plot shows the progression of the pan (orange line) and core (purple line) genomes as more genomes are added for analysis. The parameter ’b’ = 0.17 indicates the pan-genome is still open but may be closed soon. The pan-genome is still open, as the new additional genome significantly increases the total repertoire of genes. Extrapolation of the curve indicates that the gene families in pan-genome increased from 2131 to 2838, and those in core genome decreased from 1876 to 1734. (b) Flower plot shows the numbers of core genes (inner circle), accessory genes (middle circle), and unique genes (outer circle). (c) Phylogenetic tree based on the pan-genome. (d) Phylogenetic tree based on the core genome.

To better understand the phylogenetic relationship and bacterial evolution, we performed a pan-genome analysis of seven publicly available WGS of *P. multocida* strains with our isolates (Figure-7, Table-S1). The evolution of the pan and core genome is presented in Figure-7. In each new genome of *P. multocida*, the number of gene families in the pan-genome increased from 2131 to 2838 (Figures-7a), and gene families in the core genome decreased from 1876 to 1734 (Figure-7b). The phylogeny based on the pan-genome demonstrated that PM4 and PM7 are closer to strain Razi_Pm001, forming a clade with other strains C48-1 and HN06 (Figure-7c). Conversely, core genome-base phylogeny analysis showed that both Razi_Pm001 and ATCC 2095 strains of *P. multocida* clustered more closely with the study (PM4 and PM7) strains forming the same clade (Figure-7d). These findings suggest the diverse genetic evolution of the pan and core genomes in different *P. multocida* strains [87]. In this study, the unique genes of each *P. multocida* strain exhibited a wide distribution, ranging from 17 (0.6%) to 100 (3.5%). We found 90 unique genes in our isolates (Table-S2). These unique genes are found under relaxed mutation pressure, and might have an association with the pathogenicity, virulence, and AMR [88], in PM4 and PM7 strains, though being type B:2, to cause FC in layer birds. These genes enable the bacteria to transfer benefits to themselves through the horizontal gene transfer, thereby enhancing symbiosis and adaptation of the bacteria to the host, and subsequent onset of pathogenic episodes [89]. However, further investigation is necessary to prove the association of these genes with pathogenicity of FC in laying birds.

## Conclusion

This study reports the genetic diversity and genomic potentials of *P. multocida* strains isolated from the FC outbreaks in commercial layer farms of Bangladesh. In addition to conventional methods such as cultural and biochemical test, identification of the isolated organisms as *P. multocida* was confirmed by molecular approach, that is, *P. multocida* species-specific gene (*kmt1*)-based PCR, RAPD-PCR, ribosomal gene (16S rRNA) sequencing, and finally *P. multocida* genotype B: L2:ST122PCR was confirmed through cutting-edge WGS technology. Biochemical, molecular typing, and *16S rRNA* gene sequencing confirmed 22 isolates as *P. multocida* and grouped them into two major biotypes and RAPD profiles. The *in vitro* resistance profiling (antimicrobial susceptibility tests and BF assays) showed that majority of the *P. multocida* strains were multidrug-resistant and SBF. Alarmingly, most of the tested antimicrobial agents currently available remained resistant to *P. multocida* strains with the exception of colistin sulfate which was found as the most effective antimicrobial agent for treatment, prevention and control of FC in Bangladesh. The comprehensive annotations of the complete genomes of *P. multocida* strains provided valuable insights on the genomic features, including genome size, GC content, coding and non-coding regions along with the identification of several VFGs and ARGs. The pan-genome analysis identified several unique genes involved in basic metabolism, pathogenicity, virulence and AMR, and thus, implicating their survival fitness and host adaptation to causing FC. This study has opened up an avenue for further research on elucidating the mechanisms behind the molecular pathogenesis of *P. ­multocida* strains to cause FC in avian hosts with subsequent development of effective preventive and therapeutic strategies using an *in vivo* an animal-model.

## Data availability

Complete genome sequences of PM4 and PM7 are available in the NCBI GenBank database under the accession numbers CP052764 (BioSample: SAMN14639261) and CP052765 (BioSample: SAMN14639262), respectively, in the BioProject: PRJNA626386.

## Supplementary data

Supplementary data (Figures-S1-S3 and Tables-S1-S3) can be available from the corresponding author on reasonable request.

## Authors’ Contributions

OS: Carried out the studies (sampling, laboratory experiments, molecular, and data analysis). MRI and MSR: Performed the analyses and drafted the initial manuscript. MNH: Critically reviewed and interpreted the results and edited the entire manuscript. MAH, and MS: Developed the hypothesis, supervised the work, and critically reviewed the final manuscript. Finally, all authors read and approved the final manuscript.
